# Whole-genome characterization of seven multidrug-resistant *Neisseria gonorrhoeae* isolates from a single tertiary center in Beijing

**DOI:** 10.3389/fmicb.2026.1882940

**Published:** 2026-07-15

**Authors:** Jia-Hao Qin, Qiu-Min Lyu, Xiu-Ying Zhao, Lin Liu, Nan Xiao

**Affiliations:** 1 College of Basic Medical Sciences, Dalian University, Dalian, China; 2 Department of Laboratory Medicine, Beijing Tsinghua Changgung Hospital, School of Clinical Medicine, Tsinghua Medicine, Tsinghua University, Beijing, China

**Keywords:** multidrug resistance, *Neisseria gonorrhoeae*, *penA* 60.001, phylogenetic analysis, whole-genome sequencing

## Abstract

**Background:**

To characterize the whole-genome features of *Neisseria gonorrhoeae* clinical isolates collected from a tertiary medical institution in Beijing, with a focus on the genomic basis of ceftriaxone non-susceptibility and multidrug resistance.

**Methods:**

Clinical isolates were collected from April 2023 to November 2024. Of 14 collected isolates, seven were successfully subcultured after revival and included in subsequent analyses. Minimum inhibitory concentrations (MICs) were determined by the Etest method. Whole-genome data were obtained using a combination of second- and third-generation sequencing technologies. The isolates were combined with global and Chinese reference datasets to construct a core-genome single-nucleotide polymorphism (core-SNP) phylogenetic tree. Chromosomal resistance-associated mutations and plasmid characteristics were subsequently analyzed.

**Results:**

The seven isolates displayed genomic diversity at the whole-genome level. Four isolates (8087, 8423, 8461, and 8801) carried *penA* 60.001 and belonged to distinct sequence types, including ST7365, ST8123, and ST7367. One additional isolate (8726) carried *penA* 273.001; both alleles encode PBP2 proteins sharing the core substitutions A311V, I312M, V316T, and T483S. All five isolates were non-susceptible to ceftriaxone (MIC 0.25–0.5 mg/L). Ceftriaxone non-susceptibility was associated with the co-occurrence of mutations at core *penA* positions and additional mutations in *porB* and *ponA*, with an *mtrR* mutation present in one isolate. Plasmid collinearity analysis revealed that several multidrug-resistant isolates simultaneously harbored an intact conjugative plasmid and an African-type resistance plasmid carrying *bla*_TEM-1_.

**Conclusion:**

The multidrug-resistant phenotype of *Neisseria gonorrhoeae* results from the co-existence of chromosomal multi-locus mutations and resistance plasmids. The *penA* 60.001 isolates in this study did not originate from a single source. This allele appeared in multiple local clonal lineages. This pattern is consistent with horizontal gene transfer of this resistance determinant into multiple endemic lineages.

## 1 Introduction

Gonorrhea is a bacterial sexually transmitted infection caused by *Neisseria gonorrhoeae (N. gonorrhoeae)*. It remains widespread globally and continues to impose substantial burden on public health systems. The World Health Organization (WHO) estimated approximately 82.4 million new gonorrhea infections among individuals aged 15–49 years in 2020 (World Health Organization, 2025a). In China, 96,313 gonorrhea cases were reported in 2022 (Zhu et al., 2024). *Neisseria gonorrhoeae* has progressively developed resistance to penicillins, tetracyclines, fluoroquinolones, and oral cephalosporins (Shaskolskiy et al., 2022). Ceftriaxone currently serves as the first-line treatment recommended by both domestic and international guidelines (Zhu et al., 2024; Workowski et al., 2021), and ceftriaxone non-susceptibility has become a central challenge in gonorrhea management (World Health Organization, 2025b).

The molecular basis of ceftriaxone resistance primarily involves mosaic *penA* allele mutations, which reduce the binding affinity of the encoded penicillin-binding protein 2 (PBP2) for the drug (López-Argüello et al., 2024). These mutations act synergistically with *mtrR*-mediated efflux pump upregulation, *porB* porin mutations, and *ponA* mutations to establish a multi-mechanism resistance profile (Lindberg et al., 2007). Among these, the *penA* 60.001 allele—characterized by multiple amino acid substitutions including A311V and T483S—is a well-characterized allele associated with ceftriaxone resistance (Lin et al., 2016; Caméléna et al., 2024; Chen et al., 2019), and represents a key molecular target in global ceftriaxone resistance surveillance (Adamson et al., 2024).

In 2015, Japan first reported the ceftriaxone-resistant clone FC428 carrying *penA* 60.001, defined by the genotype MLST ST1903/NG-MAST ST3435/NG-STAR ST233 (Nakayama et al., 2016). FC428-related clones subsequently spread globally, with isolates detected in Australia, Canada, Denmark, France, and Ireland (Caméléna et al., 2024; Lee et al., 2019; Golparian et al., 2018). In 2016, Beijing Ditan Hospital reported the first isolation in China of a ceftriaxone-resistant strain with a genotype consistent with FC428 (Chen et al., 2019), and FC428-related strains have since been reported in multiple Chinese cities (Zhu et al., 2024). Surveillance data from Nanjing showed a year-on-year increase in the proportion of *penA* 60.001 isolates among local *N. gonorrhoeae* strains between 2017 and 2020 (Zhao et al., 2017). Data from Hangzhou further revealed that the *penA* 60.001 allele had begun transferring into locally circulating non-FC428 background strains (Yang et al., 2024), indicating that this resistance determinant may be disseminating into non-FC428 endemic lineages.

Recent whole-genome sequencing (WGS)-based molecular epidemiological studies on *N. gonorrhoeae* in Beijing remain limited (Peng et al., 2019; Wang et al., 2023). Consequently, the current transmission status of the FC428 clone in this region and the genomic diversity of resistant strains have not been systematically characterized. In this study, *N. gonorrhoeae* isolates collected from a tertiary medical institution in Beijing were analyzed in the context of global and Chinese reference genomes, to provide genomic evidence to support optimization of clinical treatment strategies and antimicrobial resistance surveillance in Beijing.

## 2 Materials and methods

### 2.1 Data collection

*Neisseria gonorrhoeae* clinical isolates were collected from a tertiary medical institution in Beijing between April 2023 and November 2024. Relevant clinical information, including patient sex, age, and specimen source site, was retrospectively collected for all isolates.

### 2.2 *Neisseria gonorrhoeae* isolation

Specimen types included urine and urethral discharge. All specimens were simultaneously inoculated onto chocolate agar and Columbia blood agar (OXOID Ltd., Thermo Fisher Scientific, Hampshire, UK) and incubated at 37 °C with 5% CO₂ for 18–24 h. Suspect colonies (grayish-white, approximately 0.5–1 mm in diameter) were selected and identified to species level by matrix-assisted laser desorption/ionization time-of-flight mass spectrometry (MALDI-TOF MS) (Bruker Corporation, Bremen, Germany). Confirmed *N. gonorrhoeae* isolates were cryopreserved and assigned sequential accession numbers according to the laboratory strain repository numbering system. A total of 14 clinical isolates were collected during the study period; of these, 7 were successfully subcultured after revival and were identified as *N. gonorrhoeae* by 16S rRNA and *porA* sequencing (Weisburg et al., 1991; Feavers and Maiden, 1998; Hjelmevoll et al., 2006; Unemo et al., 2005), and were included in subsequent analyses. Failure of revival among the remaining seven isolates was attributed to loss of bacterial viability during cryopreservation, which is a recognized technical challenge with *N. gonorrhoeae* given its fastidious growth requirements and sensitivity to storage conditions.

### 2.3 Antibiotic susceptibility testing (AST)

*In vitro* antimicrobial susceptibility of the seven *N. gonorrhoeae* clinical isolates was determined by the Etest method, with minimum inhibitory concentrations (MIC) values measured for seven antimicrobial agents: penicillin (PEN), ceftriaxone (CRO), cefepime (FEP), tetracycline (TCY), ciprofloxacin (CIP), levofloxacin (LVX), and azithromycin (AZM). Cryopreserved isolates were revived and subcultured onto Gonococcal (GC) agar (Guangzhou Detgerm Ltd., Guangdong, China) at 37 °C with 5% CO₂ for 18–24 h. Single colonies were picked and suspended to a 0.5 McFarland standard, then spread evenly onto GC agar plates. Etest strips (Wenzhou Kangtai Biotechnology Co., Ltd. Zhejiang, China) were applied, and MIC values were read after incubation at 37 °C with 5% CO₂ for 18–24 h.

Ceftriaxone susceptibility was interpreted using EUCAST Clinical Breakpoint Tables (v16.0) (The European Committee on Antimicrobial Susceptibility Testing, 2026), with non-susceptibility defined as MIC > 0.125 mg/L; this threshold is consistent with WHO GASP and the majority of WGS-based epidemiological studies on ceftriaxone-resistant *N. gonorrhoeae* (Wi et al., 2017). Penicillin, ciprofloxacin, tetracycline, and azithromycin were interpreted using CLSI M100 (36th edition) (Clinical and Laboratory Standards Institute, 2026), as CLSI provides *N. gonorrhoeae*-specific breakpoints for these four agents that are not equivalently defined under EUCAST for routine clinical reporting in China (Zhu et al., 2024). For azithromycin specifically, CLSI M100 (36th edition) defines only a susceptibility breakpoint (MIC ≤ 1 mg/L) without a resistant category; a MIC of ≥ 2 mg/L was therefore applied as the resistance threshold, in accordance with the CLSI epidemiological cutoff value (ECV) (Kersh et al., 2020). Levofloxacin and cefepime were not categorically interpreted, as no *N. gonorrhoeae*-specific breakpoints are established for either agent under EUCAST v16.0 or CLSI M100 (36th edition); MIC values for both agents are reported without S/I/R classification (The European Committee on Antimicrobial Susceptibility Testing, 2026; Clinical and Laboratory Standards Institute, 2026). Cefepime was included as an exploratory agent to assess cross-resistance patterns among extended-spectrum cephalosporins and is not a standard treatment option for *N. gonorrhoeae* infections in clinical practice (Workowski et al., 2021; Schörner et al., 2022). *N. gonorrhoeae* reference strain ATCC 49226 was used as the quality control strain.

Multidrug resistance (MDR) was defined as acquired non-susceptibility to at least one antimicrobial agent in three or more antimicrobial categories, following the criteria proposed by Magiorakos et al. (2012). The antimicrobial categories evaluated were: penicillins (penicillin), extended-spectrum cephalosporins (ceftriaxone), fluoroquinolones (ciprofloxacin), macrolides (azithromycin), and tetracyclines (tetracycline). The resistance threshold applied for azithromycin is described in Section 2.3. Levofloxacin was excluded from MDR classification as no *N. gonorrhoeae*-specific breakpoints exist under either EUCAST v16.0 or CLSI M100 (36th edition). Cefepime was similarly excluded, as no species-specific breakpoints are established for this drug–organism combination under either standard.

### 2.4 Whole-genome sequencing, *De novo* assembly, and sequence alignment

All seven *N. gonorrhoeae* clinical isolates underwent whole-genome sequencing. After 18–24 h of culture, colonies were harvested and genomic DNA was extracted using the QIAamp DNA Mini Kit (Qiagen, Germany). DNA integrity, purity, and concentration were assessed by agarose gel electrophoresis, NanoDrop (Thermo Fisher Scientific, USA), and Qubit fluorometer (Thermo Fisher Scientific, USA) prior to sequencing. Short-read sequencing was performed on an Illumina MiSeq platform (Illumina, San Diego, USA) with 150 bp paired-end reads, and long-read sequencing was performed on a MinION platform (Oxford Nanopore Technologies, Oxford, UK). Complete genome assemblies were generated using Unicycler (v0.5.1) (Wick et al., 2017), run with default parameters in hybrid mode, using short reads for initial graph construction and long reads for repeat resolution and circularisation. No minimum contig length filter was applied post-assembly. Assembly quality metrics, including genome completeness, N50, and sequencing depth for each isolate, are summarized in Supplementary Table 1.

Genome sequences were submitted to the PubMLST database (https://pubmlst.org/; last accessed 18 December 2025) for multilocus sequence typing (MLST), *Neisseria gonorrhoeae* Multi-Antigen Sequence Typing (NG-MAST), and *Neisseria gonorrhoeae* Sequence Typing for Antimicrobial Resistance (NG-STAR) classification. Resistance-associated mutations were also identified through PubMLST, including the mutational status of *penA* (mosaic allele type and key amino acid substitutions), *porB* (*penB* mutations), *ponA*, *gyrA*, *parC*, 23S rRNA, and *mtrR*. Plasmid replicon screening was performed against the PlasmidFinder database (https://cge.food.dtu.dk/services/PlasmidFinder/; last accessed 18 December 2025).

### 2.5 Phylogenetic analysis

To evaluate the phylogenetic relationships of the local isolates, a total of 207 complete *N. gonorrhoeae* genome assemblies with a release date between 2015 and 2025 were identified in the NCBI Genome database (Assembly level: Complete; https://www.ncbi.nlm.nih.gov/; last accessed 18 December 2025). Two assemblies flagged as contaminated were excluded, yielding a final reference dataset of 205 genomes (Supplementary Table 2). This dataset comprised all complete assemblies meeting the above criteria, including the FA1090 and FC428 reference genomes and isolates from multiple countries and assembly release years, and was used to provide broad global phylogenetic context for the seven local isolates. A reference dataset comprising 168 Chinese *penA* 60.001 isolates (Supplementary Table 3) was downloaded from the National Genomics Data Center (NGDC; https://ngdc.cncb.ac.cn/gsa) (Zhang et al., 2002).

For whole-genome phylogenetic analysis, FA1090 (NC_002946.2) (Supplementary Table 3) and FC428 (NZ_AP018377) (Supplementary Table 4) were used as reference genomes. Whole-genome read mapping and variant calling for the seven local isolates and reference strains were performed using Snippy (v4.6.0; https://github.com/tseemann/snippy) with default parameters (minimum mapping quality 60, minimum base quality 13, minimum read depth 10, minimum fraction of reads supporting a variant 0.9). Recombinant regions in the core-genome single-nucleotide polymorphism (core-SNP) alignment matrix were identified and masked using Gubbins (v3.4.1) with RAxML (raxmlHPC v8.2.12, GTRGAMMA model) as the internal tree builder and a maximum of five iterations, retaining only vertically inherited SNP sites for downstream analysis (Croucher et al., 2015). Recombination detection was performed using Gubbins (v3.4.1) with default parameters, using RAxML as the internal tree builder and a maximum of five iterations. Maximum-likelihood phylogenetic trees were constructed from the recombination-filtered SNP alignment using IQ-TREE (v2.4.0) (Minh et al., 2020). The best-fit nucleotide substitution model (GTR + F + ASC + G4) was selected automatically by ModelFinder under the Bayesian Information Criterion (BIC). Branch support was evaluated using 1,000 ultrafast bootstrap replicates (UFBoot2). Tree topology visualization and metadata integration were performed using the tvBOT online platform^1^ (Xie et al., 2023).

### 2.6 Sequence extraction and alignment

Reference sequences for chromosomal resistance determinants—*penA* (M32091.1), *mtrR* (Z25796), *porB* (M21289.1), *ponA* (AB727715.1), *rpsJ* (NZ_AP023069.1), 23S rRNA (NC_002946.2), *gyrA* (U08817.1), and *parC* (U08907.1)—were downloaded from GenBank. Local alignment of these targets against the hybrid-assembled genome sequences was performed using BLASTn (v2.17.0) (Camacho et al., 2009) with default parameters (word size 11, E-value threshold 10, reward/penalty +2/−3). The extracted target sequences were then aligned using MAFFT (v7.471) (Katoh and Standley, 2013) with the default progressive alignment strategy (FFT-NS-2), and mutations or substitutions at key positions were identified from the alignment results.

### 2.7 Plasmid extraction, annotation, and structural collinearity analysis

Unicycler hybrid assembly contigs were screened against the PlasmidFinder database (Carattoli et al., 2014) to identify candidate plasmid sequences carrying specific replicons. Functional annotation was performed using Bakta (v1.8.2) with the light database (version 6.0) (Schwengers et al., 2021) and default parameters. Structural collinearity alignment and visualization were carried out using Mauve (v2.4.0) (Darling et al., 2010) with default progressive alignment parameters. Reference sequences used for comparative genomic analysis were as follows: pJD1 (NC_001377.1) for the cryptic plasmid; the African-type pJD5 (U12461.1) and Asian-type pJD4 (U12462.1) for resistance plasmids; and a Dutch-type conjugative plasmid carrying *tet*(M) (GU479466.1) for the conjugative plasmid. The complete analytical workflow is presented in Supplementary Figure 1.

## 3 Results

### 3.1 Demographic profiles and revival rates of clinical isolates

A total of 14 *N. gonorrhoeae* clinical isolates were collected from the urogenital tract at Beijing Tsinghua Changgung Hospital between April 2023 and November 2024 (Table 1). Baseline patient information and specimen collection details for all 14 isolates are summarized in Table 1. Of the 14 patients, 13 were male and one was female, with ages ranging from 24 to 46 years (median 34 years). Specimen types included urine (*n* = 2), urethral discharge (*n* = 10), cervical secretion (*n* = 1), and purulent secretion (*n* = 1).

**Table 1:** Clinical and demographic information of enrolled patients and isolates.

No.	Strain ID	Collection date	Patient age (years)	Sex	Specimen type	Revival outcome
1	7972	2023/04/03	24	Male	Urine	Successful
2	8087	2023/06/20	42	Male	Urine	Successful
3	8423	2024/03/19	27	Male	Urethral discharge	Successful
4	8461	2024/04/18	30	Male	Urethral discharge	Successful
5	8533	2024/06/11	38	Male	Urethral discharge	Successful
6	8678	2024/08/10	46	Male	Urethral discharge	Failed
7	8726	2024/09/03	35	Male	Urethral discharge	Successful
8	8773	2024/10/09	32	Female	Cervical secretion	Failed
9	8787	2024/10/27	37	Male	Urethral discharge	Failed
10	8795	2024/11/03	25	Male	Urethral discharge	Failed
11	8800	2024/11/11	39	Male	Purulent Secretion	Failed
12	8801	2024/11/12	46	Male	Urethral discharge	Successful
13	8802	2024/11/14	27	Male	Urethral discharge	Failed
14	8804	2024/11/17	27	Male	Urethral discharge	Failed

Of the 14 isolates, seven (7972, 8087, 8423, 8461, 8533, 8726, and 8801) were successfully subcultured after revival from cryopreservation and were included in subsequent whole-genome sequencing analysis. The remaining seven isolates (8678, 8773, 8787, 8795, 8800, 8802, and 8804) failed revival and were excluded.

### 3.2 Ceftriaxone non-susceptibility is associated with co-occurring multi-locus chromosomal mutations in *penA*, *porB*, and *ponA*

Key resistance gene mutations and corresponding MIC values for each isolate are presented in Tables 2, 3. Five of the seven isolates (8087, 8423, 8461, 8726, and 8801) were non-susceptible to ceftriaxone (MIC 0.25–0.5 mg/L). Whole-genome analysis revealed that among the five ceftriaxone non-susceptible isolates, four (8087, 8423, 8461, and 8801) carried *penA* 60.001 and one (8726) carried *penA* 273.001. Both alleles encode PBP2 proteins sharing the core substitutions A311V, I312M, V316T, and T483S, which are the primary determinants of reduced PBP2–ceftriaxone binding affinity and underlie the non-susceptible phenotype observed in these five isolates. All five isolates also harbored chromosomal *ponA* L421P and *porB* G120K mutations; isolate 8087 additionally carried a *porB* A121D substitution. The two isolates fully susceptible to ceftriaxone (7972 and 8533) carried the *penA* 34.007 allele, which lacks the A311V and T483S substitutions, and neither harbored the *ponA* L421P mutation. Six isolates exhibited a penicillin-resistant phenotype. Five of these isolates carried the *bla*_TEM-1_ plasmid and displayed high-level penicillin resistance (MIC ranging from 16 to >32 mg/L). Isolate 8461 lacked the *bla*_TEM-1_ gene, and its penicillin MIC was 4 mg/L. Isolate 7,972 showed intermediate susceptibility to penicillin (MIC 0.38 mg/L). Sequence analysis of *mtrR* revealed heterogeneous mutation profiles across the seven isolates. Isolate 8,801 carried an *mtrR* G45D substitution, and isolate 8533 carried an *mtrR* A39T substitution. The remaining five isolates (7972, 8087, 8423, 8461, and 8726) showed no coding-sequence mutations in *mtrR* (Table 3).

**Table 2:** Chromosomal and plasmid-mediated resistance determinants of *N. gonorrhoeae* clinical isolates.

Strain ID	*penA* allele	*mtrR* mutation	*porB* mutation	*ponA* mutation	*gyrA* and *parC* mutations	23S rRNA mutation (4 copies)	Plasmid-mediated resistance
7972	34.007	—	G120K, A121D	—	*gyrA*: S91F, D95A; *parC*: S87R	—	—
8087	60.001	—	G120K, A121D	L421P	*gyrA*: S91F, D95A; *parC*: S87R	—	*bla*_TEM-1_
8423	60.001	—	G120K	L421P	*gyrA*: S91F, D95Y; *parC*: S87N	—	*bla*_TEM-1_
8461	60.001	—	G120K	L421P	*gyrA*: S91F, D95A; *parC*: S87R	—	—
8533	34.007	A39T	G120K	—	*gyrA*: S91F, D95A; *parC*: D86N	—	*bla*_TEM-1_; *tet*(M)
8726	273.001	—	G120K	L421P	*gyrA*: S91F, D95A; *parC*: S87R	—	*bla*_TEM-1_
8801	60.001	G45D	G120K	L421P	*gyrA*: S91F, D95A; *parC*: S87R	—	*bla*_TEM-1_

—, not detected. The shared core substitutions encoded by *penA* 60.001 and 273.001 (A311V, I312M, V316T, T483S) are not listed individually in the table.

**Table 3:** Antimicrobial susceptibility phenotypes of *N. gonorrhoeae* clinical isolates.

Strain ID	PEN MIC (mg/L)	CRO MIC (mg/L)	FEP MIC (mg/L)	CIP MIC (mg/L)	LVX MIC (mg/L)	TCY MIC (mg/L)	AZM MIC (mg/L)	MDR
7972	0.38 (I)	<0.064 (S)	1	16 (R)	>32	4 (R)	2 (R)	Yes
8087	>32 (R)	0.25 (NS)	2	4 (R)	4	0.5 (I)	<0.064 (S)	Yes
8423	16 (R)	0.5 (NS)	2	>32 (R)	>32	0.5 (I)	0.125 (S)	Yes
8461	4 (R)	0.5 (NS)	4	>32 (R)	>32	1 (I)	0.5 (S)	Yes
8533	24 (R)	<0.064 (S)	0.5	2 (R)	4	64 (R)	0.25 (S)	Yes
8726	>32 (R)	0.5 (NS)	2	2 (R)	8	0.5 (I)	<0.064 (S)	Yes
8801	>32 (R)	0.5 (NS)	4	16 (R)	16	4 (R)	0.5 (S)	Yes

All seven isolates were resistant to ciprofloxacin by CLSI M100 (36th edition) criteria (MIC ≥1 mg/L). Levofloxacin MICs were markedly elevated across all isolates (range 4 to >32 mg/L); however, no species-specific breakpoints for levofloxacin against *N. gonorrhoeae* are established under either EUCAST v16.0 (The European Committee on Antimicrobial Susceptibility Testing, 2026) or CLSI M100 (36th edition) (Clinical and Laboratory Standards Institute, 2026), and categorical susceptibility interpretation is not applicable for this agent (Table 3). Resistance gene analysis showed that all isolates carried dual *gyrA* mutations (S91F combined with either D95A or D95Y) alongside a single *parC* mutation (S87R, S87N, or D86N).

For tetracycline, the ribosomal protein S10 gene (*rpsJ*) V57M substitution was detected in all seven isolates. Among the six isolates without *tet*(M), tetracycline MIC values ranged from 0.5 to 4 mg/L. Isolate 8533 had a tetracycline MIC of 64 mg/L, with sequence analysis confirming the presence of a large conjugative plasmid carrying *tet*(M). Among the six *tet*(M)-negative isolates, strains 7972 and 8801 had tetracycline MICs of 4 mg/L each, higher than the remaining four (0.5–1 mg/L). Isolate 8801 co-harbored *rpsJ* V57M, *porB* G120K, and *ponA* L421P; isolate 7,972 carried dual *porB* mutations, G120K and A121D.

Regarding macrolide susceptibility, isolate 7972 was resistant to azithromycin (MIC 2 mg/L). No A2059G or C2611T substitutions were detected in any of the four 23S rRNA allele copies across the seven isolates, and no single-nucleotide deletion (ΔA) was identified in the 13-bp inverted repeat of the *mtrR* promoter region. Sequence analysis of *mtrD* in isolate 7,972 identified S821A and K823E substitutions within the drug-binding PC2 subdomain, and *rplD* analysis revealed V125A, A147G, and R157Q co-substitutions in the encoded 50S ribosomal protein L4. Both the *mtrD* K823E substitution and the *rplD* V125A/A147G/R157Q combination were absent in the remaining six azithromycin-susceptible isolates.

All seven isolates met the criteria for multidrug resistance, with non-susceptibility spanning three to four antimicrobial categories (Table 3). Isolate 8,801 exhibited the broadest resistance profile, with non-susceptibility across four categories: penicillins, extended-spectrum cephalosporins, fluoroquinolones, and tetracyclines. Isolate 7,972 represented a distinct resistance pattern, with susceptibility to ceftriaxone but resistance to fluoroquinolones, azithromycin, and tetracyclines simultaneously.

All seven failed isolates were collected between August and November 2024. Based on available susceptibility data obtained by disk diffusion, the proportion of ceftriaxone non-susceptible isolates among the excluded strains (2/7, 28.6%) was identical to that observed among the included isolates by the same method (2/7, 28.6%). However, MIC confirmation was not available for the excluded isolates, and disk diffusion may underestimate the true prevalence of ceftriaxone non-susceptibility at borderline MIC values (Supplementary Table 4).

Categorical interpretation of ceftriaxone MICs under EUCAST v16.0 and CLSI M100 (36th edition) was concordant for six of seven isolates. The sole discordant result was observed for isolate 8,087 (MIC 0.25 mg/L), classified as non-susceptible by EUCAST v16.0 but intermediate by CLSI M100 (36th edition). For the remaining four non-susceptible isolates (MIC 0.5 mg/L; 8,423, 8,461, 8,726, and 8,801), both systems consistently identified non-susceptibility, despite differing categorical labels (NS vs. R) (Table 4).

**Table 4:** Comparison of ceftriaxone MIC categorical interpretations under EUCAST v16.0 and CLSI M100 (36th edition).

Strain ID	CRO MIC (mg/L)	EUCAST v16.0 (NS: MIC > 0.125 mg/L)	CLSI M100 (S: MIC ≤ 0.12 mg/L; I: MIC = 0.25 mg/L; R: MIC ≥ 0.5 mg/L)	Concordance
7972	<0.064	S	S	Yes
8087	0.25	NS	I	No
8423	0.5	NS	R	Yes
8461	0.5	NS	R	Yes
8533	<0.064	S	S	Yes
8726	0.5	NS	R	Yes
8801	0.5	NS	R	Yes

CRO non-susceptibility (NS) defined by EUCAST Clinical Breakpoint Tables v16.0 (MIC >0.125 mg/L). Breakpoints for PEN, CIP, and TCY applied according to CLSI M100 (36th edition). AZM resistance defined as MIC ≥2 mg/L per WHO GASP epidemiological cut-off; CLSI M100 (36th edition) provides only a susceptible breakpoint for azithromycin (MIC ≤1 mg/L) without a defined resistant category. No species-specific breakpoints exist for FEP or LVX against *N. gonorrhoeae* under either EUCAST v16.0 or CLSI M100 (36th edition); MIC values for these agents are reported without categorical interpretation. R = resistant; NS = non-susceptible (ceftriaxone only); I = intermediate; S = susceptible; MDR = Multidrug Resistance.

EUCAST break point for non-susceptibility threshold for ceftriaxone is MIC >0.125 mg/L. CLSI M100 (36th edition) break points for ceftriaxone: S ≤ 0.12 mg/L, I = 0.25 mg/L, R ≥ 0.5 mg/L. Concordance was defined as agreement in clinical interpretation category between the two systems, with susceptible (S) contrasted against all non-susceptible categories (I/NS/R). The sole discordant result was observed for isolate 8,087 (MIC 0.25 mg/L), classified as non-susceptible by EUCAST v16.0 but intermediate by CLSI M100 (36th edition). For the remaining four non-susceptible isolates (MIC 0.5 mg/L), both systems consistently identified non-susceptibility, despite differing categorical labels (NS vs. R).

### 3.3 Polyphyletic distribution of *penA* 60.001 isolates across global phylogenetic clades, independent of FC428

**Figure 1:**
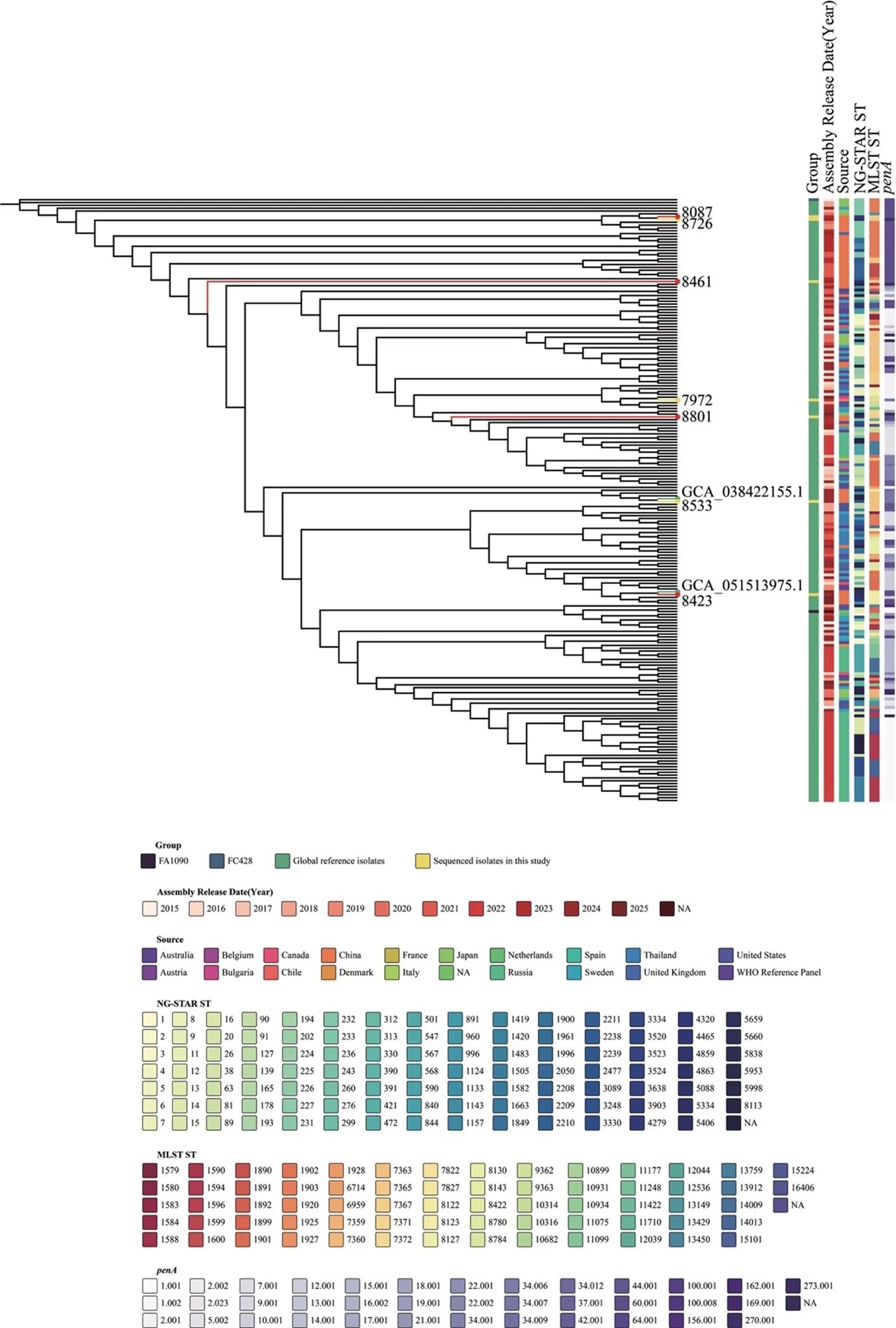
Whole-genome core-SNP phylogenetic tree of *N. gonorrhoeae* isolates in a global genomic context. Colored circles with numerical labels at branch tips identify the seven clinical isolates sequenced in this study (red circles: isolates carrying the *penA* 60.001 allele; yellow circles: non-*penA* 60.001 isolates). Green circles with numerical labels indicate Chinese reference strains clustering closely with local isolates (GCA_051513975.1 and GCA_038422155.1). Annotation tracks surrounding the tree display strain metadata from inner to outer as follows. The innermost “Group” track uses colored squares to indicate strain origin: yellow for isolates from this study, green for global reference strains from GenBank, and black and blue for the international reference strains FA1090 and FC428, respectively. Subsequent tracks indicate isolation year (2015–2024), country/region of origin, molecular typing (NG-STAR ST and MLST ST), and *penA* mutation type.

The initial core-SNP alignment matrix comprised 21,465 polymorphic sites. After recombination masking with Gubbins, 14,360 parsimony-informative sites were retained for tree construction. As shown in Figure 1, the seven local *N. gonorrhoeae* isolates were distributed across distinct clades in the whole-genome phylogenetic tree and did not form a monophyletic clade.

The four local isolates carrying *penA* 60.001 (8087, 8423, 8461, and 8801) exhibited a polyphyletic distribution across the global phylogeny, belonging to three distinct MLST backgrounds—ST7365, ST8123, and ST7367—and were scattered across different global clades, with none clustering together with the original FC428 clone (MLST ST1903). Isolate 8423 (ST8123) formed a tight cluster with a Chinese ST8123 reference strain also carrying *penA* 60.001 (GCA_051513975.1). Isolate 8087 (ST7365) grouped within the same clade as the local isolate 8726 (ST7365), the latter carrying *penA* 273.001.

Isolate 8533 (ST7363, *penA* 34.007) clustered with domestic reference strains of the same sequence type (e.g., GCA_038422155.1), while isolate 7972 (ST7372, *penA* 34.007) was positioned on a separate, distantly related branch of the phylogenetic tree.

### 3.4 Phylogenetic clustering of local *penA* 60.001 isolates with endemic Chinese clonal lineages

**Figure 2:**
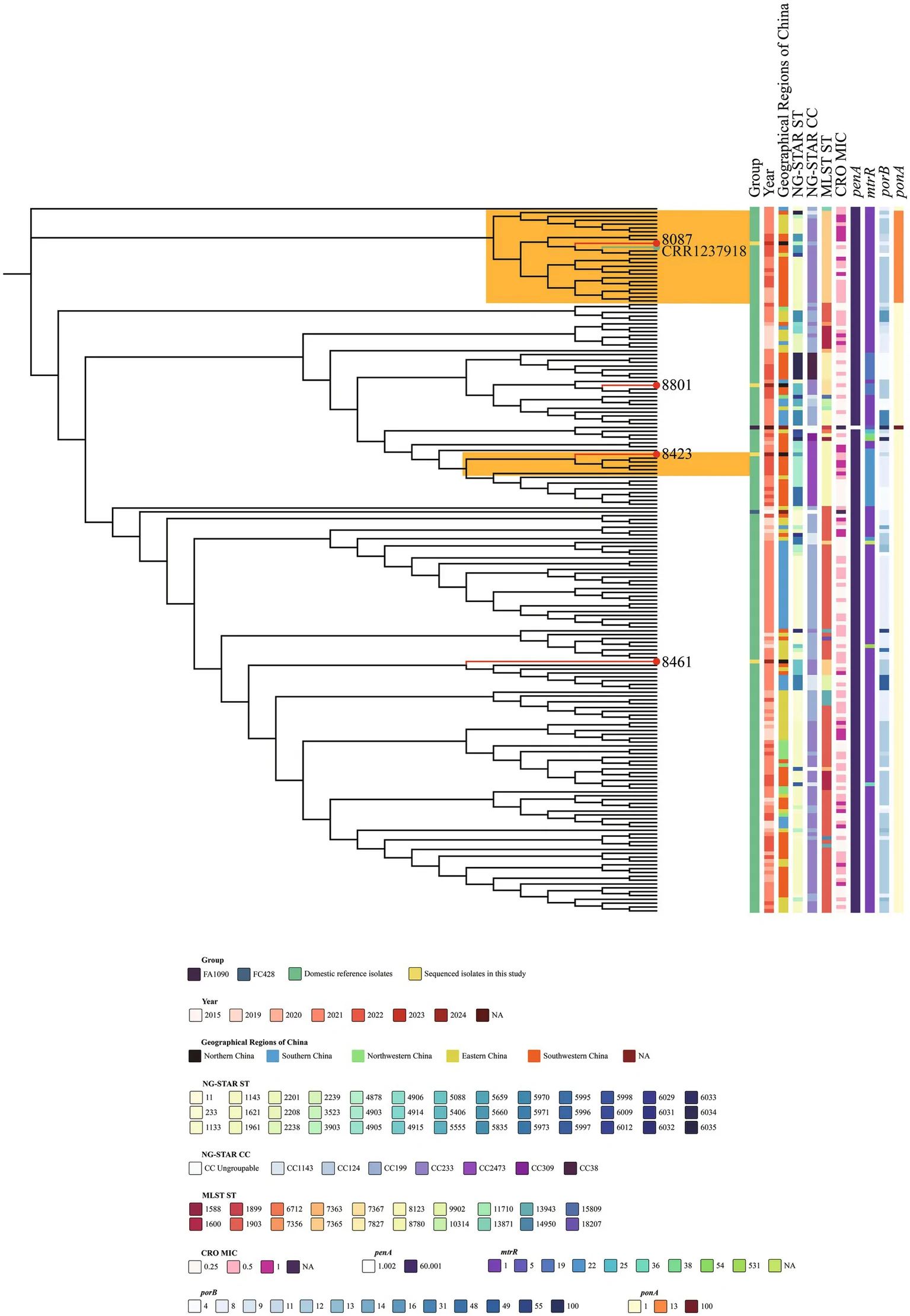
Whole-genome core-SNP phylogenetic tree of *penA* 60.001-carrying *N. gonorrhoeae* isolates in a Chinese genomic context. Red circles with numerical labels at branch tips identify the four clinical isolates carrying the *penA* 60.001 allele sequenced in this study. Green circles with numerical labels indicate Chinese reference strains clustering closely with local isolates (CRR1237918). Yellow boxes highlight the specific clades containing local isolates 8087 (ST7365) and 8423 (ST8123), emphasizing their high phylogenetic similarity to reference strains from particular domestic geographic regions (southwestern and eastern China). Annotation tracks surrounding the tree display strain metadata from inner to outer as follows. The innermost “Group” track uses colored squares to indicate strain origin: yellow for isolates from this study, green for domestic reference strains from NGDC, andblack and blue for the international reference strains FA1090 and FC428, respectively. Subsequent tracks indicate isolation year (2015–2024), Chinese geographic region of origin, molecular typing (NG-STAR ST, NG-STAR CC, and MLST ST), ceftriaxone phenotype (CRO MIC), and key resistance determinant status (including *penA* allele type and mutation profiles of *mtrR, porB,* and *ponA*).

The alignment matrix included 184 genome sequences. After recombination masking, 7,909 vertically inherited SNP sites were retained, of which 5,250 were parsimony-informative. As shown in Figure 2, the phylogenetic tree of *penA* 60.001-carrying *N. gonorrhoeae* from China comprised multiple clonal clusters composed of distinct sequence types. The FC428 clone (ST1903/CC199) constituted only one of these clades, while non-ST1903 lineages—including ST7365, ST8123, and ST7367—each formed multiple independent clades that collectively represented a substantial proportion of the tree.

The four locally isolated *penA* 60.001 strains were distributed across three distantly related branches. Isolate 8423 (ST8123) clustered with high phylogenetic similarity alongside an ST8123 clonal cluster predominantly comprising strains isolated from Southwestern China in 2022, with a minimum SNP distance of 46 to the closest reference strain (CRR1238094). Isolate 8087 (ST7365) was positioned adjacent to an ST7365 strain isolated from Eastern China in 2022 (CRR1238016; SNP distance = 47), and was placed within a larger clade containing multiple ST7365 strains from Southwestern and Eastern China. Isolates 8461 (ST7365) and 8801 (ST7367) were each located at the terminal branches of their respective independent clades, with minimum SNP distances of 52 (CRR1238071) and 61 (CRR1238087) to their closest reference strains, respectively. Pairwise SNP distances among the four local *penA* 60.001 isolates ranged from 321 (8,087 vs. 8,461) to 1,638 (8,087 vs. 8,423), confirming their phylogenetic independence (Supplementary Table 5).

### 3.5 Co-existence of conjugative and resistance plasmids in multidrug-resistant isolates

**Figure 3:**
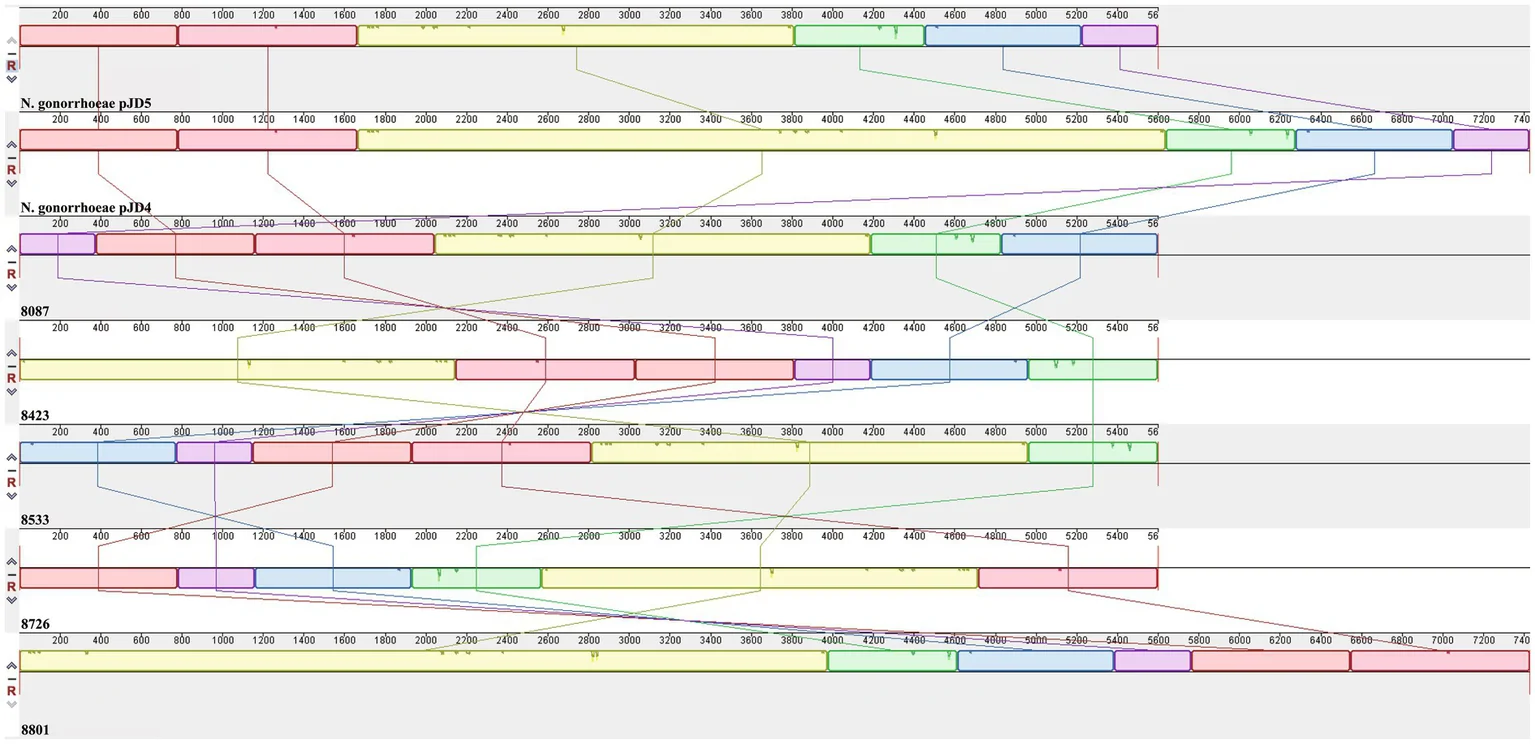
Collinearity analysis of resistance plasmids in *N. gonorrhoeae* clinical isolates. African-type pJD5 and Asian-type pJD4 were used as reference sequences. Blocks of the same color represent highly homologous collinear regions, and connecting lines indicate the positional correspondence of shared sequences across plasmids.

Plasmid annotation results (Table 5; Supplementary Table 6) showed that all seven isolates harbored a cryptic plasmid of approximately 4.2 kb. In addition, five isolates (8087, 8423, 8461, 8533, and 8726) carried a large conjugative plasmid with an intact conjugative transfer backbone (Supplementary Figure 2). Structural collinearity visualization of resistance plasmids (Figure 3) revealed that five isolates (8087, 8423, 8533, 8726, and 8801) harbored resistance plasmids, all of which contained an intact *β*-lactamase gene *bla*_TEM-1_. The resistance plasmids in isolates 8087, 8423, 8533, and 8726 were approximately 5.6 kb in length and showed high collinearity with the African-type plasmid pJD5, whereas the resistance plasmid in isolate 8801 was 7.4 kb and corresponded in topology to the Asian-type plasmid pJD4.

**Table 5:** Plasmid profiles of the *N. gonorrhoeae* clinical isolates.

Strain ID	Type of plasmid		
	Conjugative plasmid	Resistance plasmid (Type)	Cryptic plasmid
7972	—	—	√
8087	√	√ (pJD5)	√
8423	√	√ (pJD5)	√
8461	√	—	√
8533	√	√ (pJD5)	√
8726	√	√ (pJD5)	√
8801	—	√ (pJD4)	√

“*√*,” present; “—,” absent. All resistance plasmids harbor the *bla_TEM-1_* gene.

## 4 Discussion

This study characterized the phylogenetic relationships and resistance mechanisms of *N. gonorrhoeae* isolates from a single tertiary medical institution in Beijing using whole-genome sequencing. The data indicate that ceftriaxone non-susceptible isolates carrying the *penA* 60.001 allele have emerged among the patient population at this center. The four *penA* 60.001 isolates were distributed across distinct sequence types—ST7365, ST8123, and ST7367—and did not cluster with the original FC428 clone (ST1903); instead, each formed independent clusters with circulating strains from Southwestern and Eastern China. Earlier reports attributed the global dissemination of *penA* 60.001 primarily to clonal expansion of FC428 (Nakayama et al., 2016; Lee et al., 2019). However, the phylogenetic topology observed in this study suggests that *penA* 60.001 has been horizontally transferred into multiple dominant indigenous clonal lineages in China (Xiu et al., 2025; van Hal et al., 2026), and that the non-susceptible isolates identified here are not derived from a single source.

This study further examined the relationship between resistance phenotypes and resistance determinants. Analysis of the genomic and phenotypic data showed that mosaic *penA* recombination was associated with the ceftriaxone non-susceptible phenotype observed in local isolates (Lindberg et al., 2007). Notably, all five non-susceptible isolates (8087, 8423, 8461, 8726, and 8801) co-harbored *penA*, *porB*, and *ponA* mutations. Of these, isolate 8801 additionally carried an *mtrR* G45D mutation, while the remaining four non-susceptible isolates had no detectable *mtrR* mutations. This indicates that *mtrR* mutation is not required for local isolates to reach ceftriaxone MICs of 0.25–0.5 mg/L. Instead, the non-susceptible phenotype is mainly driven by mutations in *penA*, *porB*, and *ponA.* The *mtrR* G45D mutation in isolate 8,801 upregulates efflux pump expression (Chen et al., 2019). This mutation acts synergistically with target-site alterations to contribute to the multidrug-resistant phenotype of this isolate (Lindberg et al., 2007).

For other antimicrobial classes, whole-genome data revealed distinct resistance mutation profiles. All seven isolates carried *gyrA* and *parC* target mutations conferring fluoroquinolone resistance, consistent with the molecular mechanisms of fluoroquinolone resistance in *N. gonorrhoeae* described in the literature (Collins et al., 2024). Isolates carrying *gyrA* D95Y (rather than D95A) combined with *parC* S87N (8423 and 8461) had ciprofloxacin MICs exceeding 32 mg/L, higher than isolates with other *gyrA*/*parC* mutation combinations, suggesting that specific mutation combinations may influence the level of resistance conferred (David et al., 2024). For tetracycline, all seven isolates carried the *rpsJ* V57M substitution, which represents the foundational genetic background for chromosomally mediated resistance. Among the six isolates without *tet*(M), isolates 8801 and 7972 had MICs of 4 mg/L each, higher than the remaining four (0.5–1 mg/L). Compared with isolate 8423, isolate 8801 additionally harbored an *mtrR* G45D mutation on a background of *rpsJ* V57M and *porB* G120K. This is consistent with previous findings that *rpsJ* mutations, in combination with porin alterations and efflux pump upregulation, can mediate higher-level chromosomal tetracycline resistance (Hu et al., 2005). Isolate 7972 had a tetracycline MIC of 4 mg/L despite lacking *mtrR* mutations, and carried dual *porB* mutations G120K and A121D. Isolates 7972 and 8087 share the same known tetracycline resistance determinants (*rpsJ* V57M, *porB* G120K and A121D) and both lack *mtrR* mutations and *tet*(M) plasmids. The azithromycin-resistant phenotype of isolate 7972 (MIC 2 mg/L) is attributable to two candidate molecular mechanisms identified by genomic analysis. First, *mtrD* sequence alignment against the FA1090 reference revealed a K823E substitution within the drug-binding PC2 subdomain of MtrD, a residue whose substitution has been associated with elevated azithromycin MICs in clinical *N. gonorrhoeae* isolates (Ma et al., 2020a; Lyu et al., 2020). Second, *rplD* alignment identified three co-occurring substitutions (V125A, A147G, and R157Q) in the encoded 50S ribosomal protein L4, a combination significantly associated with azithromycin MIC ≥1 mg/L in clinical isolates. The remaining six isolates, all azithromycin-susceptible, lacked both the *mtrD* K823E substitution and the *rplD* V125A/A147G/R157Q combination. Direct functional confirmation via isogenic experiments in this genetic background was not performed, and the independent contribution of each variant cannot be fully resolved from genomic data alone (Mauffrey et al., 2024; Ma et al., 2020b).

The mechanism underlying the entry of *penA* 60.001 into non-FC428 lineages is consistent with natural transformation. *N. gonorrhoeae* is naturally competent throughout its life cycle and takes up exogenous DNA via the type IV pilus-mediated transformation machinery (Manoharan-Basil et al., 2021). The mosaic *penA* allele exhibits near-identical sequences across phylogenetically distant lineages, including the Swedish ST8130 isolate and the Hangzhou non-FC428 strains (Yang et al., 2024). This pattern of sequence conservation across divergent chromosomal backgrounds is characteristic of recent horizontal transfer rather than vertical inheritance. It should be noted, however, that this study did not directly delineate recombination tracts flanking *penA* 60.001 at the nucleotide level; the inference of horizontal gene transfer is therefore based on indirect evidence, specifically the polyphyletic distribution of an allele with near-identical sequence across genomically divergent lineages. The selective pressure driving this transfer is likely the sustained clinical use of ceftriaxone as first-line monotherapy in China. The fitness impact of *penA* 60.001 appears to be condition-dependent: Zhou et al. demonstrated that in standard *in vitro* growth conditions, isogenic mutants carrying *penA* 60.001 showed no significant growth disadvantage compared to *penA* 10.001 counterparts, though competitive fitness under *in vivo* conditions was reduced (Zhou et al., 2020). Compensatory mutations selected during infection may partially restore this cost, as shown for other mosaic *penA* alleles (Vincent et al., 2018). Together, these data suggest that *penA* 60.001 does not impose a prohibitive fitness penalty across all growth environments, which may facilitate its stable maintenance in diverse recipient lineages following horizontal transfer.

The early global dissemination of *penA* 60.001 was primarily driven by clonal expansion of ST1903 (FC428), in which resistance and strain fitness were co-optimized within a single genetic background (Lee et al., 2019; Zhou et al., 2020). The phylogenetic data from the present study are consistent with findings from Hangzhou reported by Yang et al. (2024) and the nationwide genomic surveillance by Zhang et al. (2002), which tracked *penA* 60.001-carrying strains across eight Chinese provinces from 2002 to 2022. Together, these data suggest that the dissemination pattern of *penA* 60.001 in China has shifted from single-clone expansion toward multi-lineage horizontal gene transfer (HGT). Under sustained ceftriaxone prescription pressure, strains that successfully acquire *penA* 60.001 gain a selective advantage regardless of their chromosomal background. The *penA* 60.001 allele is now detectable across multiple dominant endemic sequence types (including ST7365, ST8123, and ST7367) each forming independent clonal clusters with established local circulation. Among these, ST7365 constitutes one of the most prevalent ceftriaxone non-susceptible lineages in China (Yang et al., 2024; Zhang et al., 2002). Its high population prevalence in local gonorrhea transmission networks may provide a sufficiently large recipient pool to sustain onward transmission of the acquired resistance determinant following horizontal transfer. The co-occurrence of *penA* 60.001 across phylogenetically divergent lineages is consistent with HGT of this allele into multiple endemic backgrounds, rather than spread of a single resistant clone. This pattern is epidemiologically more concerning than single-clone expansion, because resistance is distributed across multiple genetic backgrounds and will persist even if any individual lineage declines in prevalence.

Furthermore, the isolates displayed co-existing chromosomal mutations and resistance plasmids. For tetracycline, the resistance phenotype ranged from chromosomally mediated low-to-moderate resistance to plasmid-mediated high-level resistance. Collinearity analysis showed that certain isolates (e.g., 8087 and 8423) had acquired a 5.6 kb African-type resistance plasmid carrying *bla*_TEM-1_ while simultaneously harboring multiple chromosomal mutations conferring ceftriaxone non-susceptibility. Multi-center surveillance data from China indicate that the African-type plasmid has risen rapidly in prevalence among penicillinase-producing *N. gonorrhoeae* (PPNG) in China (Zheng et al., 2015; Qin et al., 2025). Specifically, data from Guangdong Province show that its detection rate increased from 18.42% in 2013 to 91.55% in 2022. Conversely, the proportion of Asian-type plasmids declined from 81.58 to 7.58% over the same period. These trends establish the African-type plasmid as the dominant type among Chinese PPNG. This rapid expansion may be attributable to regional clonal proliferation of specific epidemic lineages. The hydrolytic activity of *bla*_TEM-1_ against ceftriaxone is negligible, and ceftriaxone resistance rates in PPNG carrying the African-type plasmid do not differ significantly from those in non-PPNG strains (Li et al., 2025). Accordingly, the ceftriaxone non-susceptible phenotype of isolates 8087 and 8423 is primarily driven by the chromosomal *penA* 60.001 allele in combination with *porB* and *ponA* mutations. Therefore, the *bla*_TEM-1_ carried on the African-type plasmid does not constitute the direct molecular basis for ceftriaxone resistance in these strains. The phenotypic data further validate this genomic feature. The five isolates carrying the *bla*_TEM-1_ gene all exhibited high-level penicillin resistance (MIC ≥ 16 mg/L), which is consistent with the typical characteristics of PPNG. In contrast, isolate 8461 lacked the *bla*_TEM-1_ plasmid, yet its penicillin MIC reached 4 mg/L. This indicates that in the absence of a resistance plasmid, the accumulation of chromosomal multi-locus mutations involving *penA* 60.001 combined with *porB* and *ponA* is sufficient to mediate chromosomally mediated penicillin resistance. In addition, isolate 8533 acquired a large conjugative plasmid carrying *tet*(M) on a background of chromosomal *rpsJ* V57M, resulting in high-level tetracycline resistance (Hu et al., 2005). The co-existence of multi-locus chromosomal mutations and horizontally transferred resistance plasmids may together confer broad-spectrum resistance to multiple clinically relevant antimicrobial agents (Mlynarczyk-Bonikowska et al., 2022).

This study has three notable limitations. First, it is a single-center study conducted at one tertiary institution in the northern part of Beijing, and the findings cannot be generalized to the broader epidemiological landscape of the region. Second, the sample size is small; only seven isolates were successfully included and sequenced, limiting the statistical power of the data. The possibility of residual selection bias in the analyzed dataset cannot be entirely excluded. Third, the observation period is short; the specimen collection window spanned only 19 months, which is insufficient to capture the long-term evolutionary dynamics of resistant clones. Multi-center, large-sample, prospective multi-center genomic surveillance studies should be carried out in future studies.

## 5 Conclusion

Using whole-genome sequencing, this study characterized the genomic features and resistance mechanisms of *N. gonorrhoeae* isolates collected from a single tertiary medical institution in Beijing, China.

The four ceftriaxone non-susceptible isolates carrying the *penA* 60.001 allele belonged to distinct sequence types (ST7365, ST8123, and ST7367) and were not derived from a single source, a finding consistent with horizontal transfer of this allele into multiple endemic clonal lineages. The multidrug-resistant phenotype of these strains was collectively determined by multi-locus chromosomal mutations in *penA*, *porB*, and *ponA*, together with resistance plasmids carrying *bla*_TEM-1_ or *tet*(M).

## Data Availability

The data presented in the study are deposited in the NCBI Sequence Read Archive (SRA) repository, accession number PRJNA1463503.

## Footnotes

1
https://www.chiplot.online/tvbot.html

## Glossary

*N. gonorrhoeae*: *Neisseria gonorrhoeae*
WHO: World Health Organization
PBP2: penicillin-binding protein 2
WGS: whole-genome sequencing;
MICs: minimum inhibitory concentrations
PEN: penicillin
CRO: ceftriaxone
FEP: cefepime
TCY: tetracycline
CIP: ciprofloxacin
LVX: levofloxacin
AZM: azithromycin
EUCAST: European Committee on Antimicrobial Susceptibility Testing
CLSI: Clinical and Laboratory Standards Institute
ECV: epidemiological cutoff value
MDR: Multidrug resistance
MLST: multi-locus sequence typing
NG-MAST: *Neisseria gonorrhoeae* Multi-Antigen Sequence Typing
NG-STAR: *Neisseria gonorrhoeae* Sequence Typing for Antimicrobial Resistance
core-SNP: core-genome single-nucleotide polymorphism
NS: non-susceptibility
PPNG: penicillinase-producing *Neisseria gonorrhoeae*
HGT: horizontal gene transfer
