# Correction: Whole-genome characterization of seven multidrug-resistant *Neisseria gonorrhoeae* isolates from a single tertiary center in Beijing

**DOI:** 10.3389/fmicb.2026.1967914

**Published:** 2026-09-01

**Authors:** Jia-Hao Qin, Qiu-Min Lyu, Xiu-Ying Zhao, Lin Liu, Nan Xiao

**Affiliations:** 1 College of Basic Medical Sciences, Dalian University, Dalian, China; 2 Department of Laboratory Medicine, Beijing Tsinghua Changgung Hospital, School of Clinical Medicine, Tsinghua Medicine, Tsinghua University, Beijing, China

**Keywords:** multidrug resistance, *Neisseria gonorrhoeae*, *penA* 60.001, phylogenetic analysis, whole-genome sequencing

There was an error in the placement of Figure 1 in the published version of this article. Specifically, the image of Figure 2 was mistakenly placed in the position of Figure 1, while the original Figure 2 remained unchanged. Consequently, the published article contained two identical Figure 2 images, and the correct Figure 1 was missing.

The corrected Figure 1 appears below.

**Figure 1:**
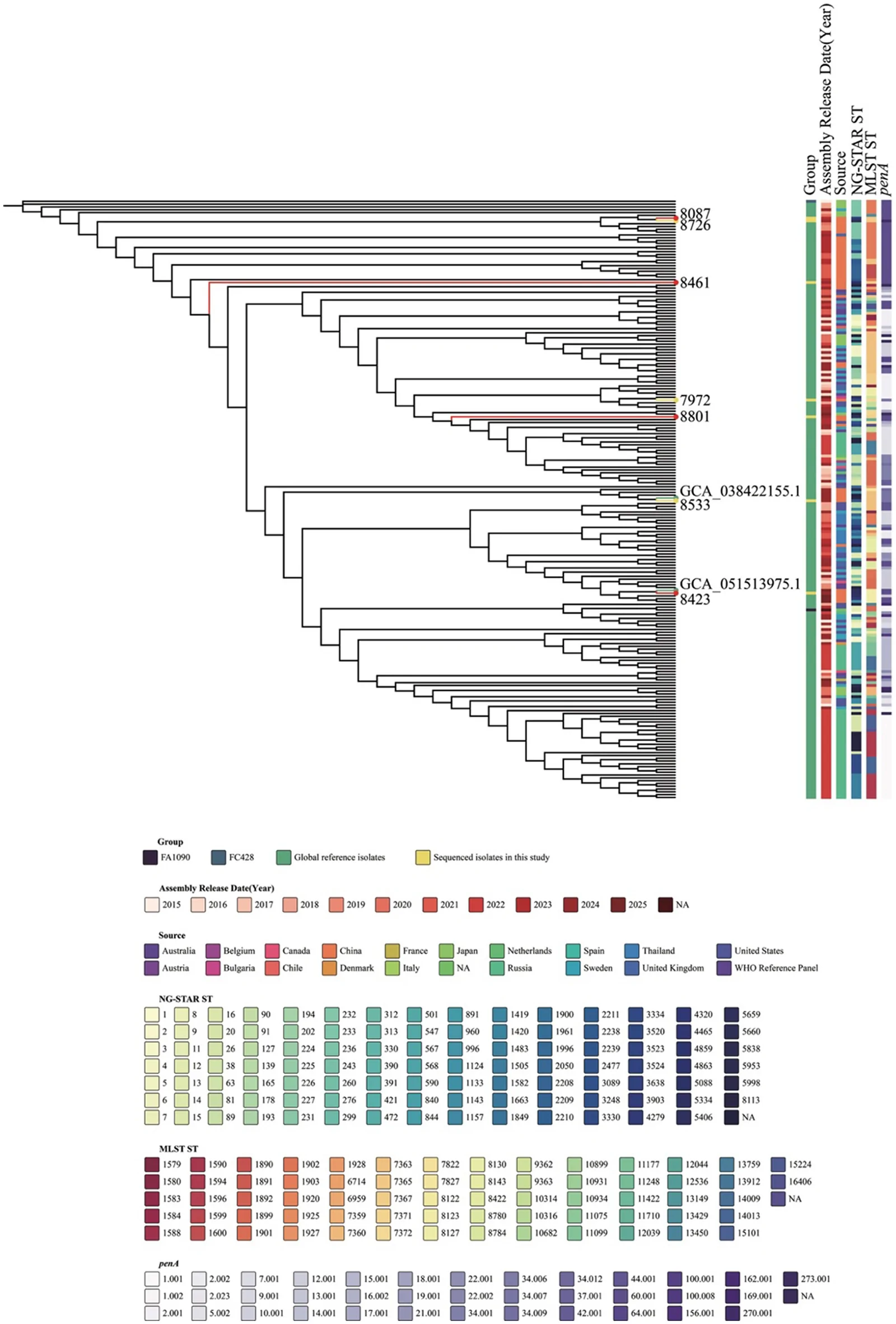
Whole-genome core-SNP phylogenetic tree of *N. gonorrhoeae* isolates in a global genomic context. Colored circles with numerical labels at branch tips identify the seven clinical isolates sequenced in this study (red circles: isolates carrying the *penA* 60.001 allele; yellow circles: non-*penA* 60.001 isolates). Green circles with numerical labels indicate Chinese reference strains clustering closely with local isolates (GCA_051513975.1 and GCA_038422155.1). Annotation tracks surrounding the tree display strain metadata from inner to outer as follows. The innermost “Group” track uses colored squares to indicate strain origin: yellow for isolates from this study, green for global reference strains from GenBank, and black and blue for the international reference strains FA1090 and FC428, respectively. Subsequent tracks indicate isolation year (2015–2024), country/region of origin, molecular typing (NG-STAR ST and MLST ST), and *penA* mutation type.

The original version of this article has been updated.

